# 1α,25(OH)_2_D_3_ attenuates IL-6 and IL-1β-mediated inflammatory responses in macrophage conditioned medium-stimulated human white preadipocytes by modulating p44/42 MAPK and NF-κB signaling pathways

**DOI:** 10.1186/s13098-019-0405-2

**Published:** 2019-01-25

**Authors:** Jingjing Zhu, Chen Bing, John P. H. Wilding

**Affiliations:** 1 Institute of Ageing and Chronic Disease, William Henry Duncan Building, University of Liverpool, 6 West Derby Street, Liverpool, L7 8TX UK; 2 Department of Endocrinology and Metabolism, The Second Affiliated Hospital of Soochow University, 1055 Sanxiang Road, Gusu District, Suzhou, 215004 People’s Republic of China; 3 Clinical Science Center, University Hospital Aintree, Longmoor Lane, Liverpool, L9 7AL UK

**Keywords:** Human white preadipocytes, Macrophages, IL-6, IL-1β, Inflammatory responses, relA, p44/42 MAPK, 1α,25(OH)_2_D_3_

## Abstract

**Background:**

Metabolic syndrome is characterized by macrophage infiltration and inflammatory responses—metaflammation in adipose tissue. IL-6 and IL-1β could mediate the inflammatory responses in macrophage stimulated-preadipocytes by modulating MAPK and NF-κB pathways. To test this hypothesis we used antibodies to block IL-6 and IL-1β action in macrophage conditioned medium (MacCM)-stimulated human white preadipocytes. Moreover, as interventions that prevent this could potentially be used to treat or prevent metabolic syndrome, and 1α,25(OH)_2_D_3_ has previously been reported to exert an anti-inflammatory action on macrophage-stimulated adipocytes, in this study we also investigated whether 1α,25(OH)_2_D_3_ could attenuate inflammatory responses in MacCM-stimulated preadipocytes, and explored the potential anti-inflammatory mechanisms.

**Methods:**

Human white preadipocytes were cultured with 25% MacCM for 24 h to elicit inflammatory responses. This was confirmed by measuring the concentrations and mRNA levels of major pro-inflammatory factors [IL-1β, IL-6, IL-8, monocyte chemoattractant protein (MCP)-1 and regulated on activation, normal T cell expressed and secreted (RANTES)] by ELISA and qPCR, respectively. IL-6 and IL-1β actions were blocked using IL-6 antibody (300 ng/ml) and IL-1β antibody (15 μg/ml), respectively. Potential anti-inflammatory effects of 1α,25(OH)_2_D_3_ were investigated by pre-treatment and treatment of 1α,25(OH)_2_D_3_ (0.01 to 10 nM) for 48 h in MacCM-stimulated preadipocytes. In parallel, western blotting was used to determine inflammatory signaling molecules including relA of the NF-κB pathway and p44/42 MAPK modified during these processes.

**Results:**

MacCM enhanced the secretion and gene expression of IL-1β, IL-6, IL-8, MCP-1 and RANTES by increasing the phosphorylation levels of relA and p44/42 MAPK in preadipocytes, whereas blocking IL-6 and IL-1β action inhibited the inflammatory responses by decreasing p44/42 MAPK and relA phosphorylation, respectively. Furthermore, 10 nM of 1α,25(OH)_2_D_3_ generally inhibited the IL-6 and IL-1β-mediated inflammatory responses, and reduced both p44/42 MAPK and relA phosphorylation in MacCM-stimulated preadipocytes.

**Conclusions:**

1α,25(OH)_2_D_3_ attenuates IL-6 and IL-1β-mediated inflammatory responses, probably by inhibiting p44/42 MAPK and relA phosphorylation in MacCM-stimulated human white preadipocytes.

**Electronic supplementary material:**

The online version of this article (10.1186/s13098-019-0405-2) contains supplementary material, which is available to authorized users.

## Background

Metabolic syndrome is a clustering of clinical findings consisting of abdominal obesity, high glucose, high triglycerides, low high-density lipoprotein cholesterol levels, and hypertension [1]. Metaflammation in adipose tissue, characterized by infiltration of macrophages, local gene expression and secretion of pro-inflammatory factors, especially IL-6 and IL-1β [2], is considered a potential factor contributing to development of the metabolic syndrome [1]. Moreover, it is notable that inflammatory signaling molecules including relA (NF-κB p65) and MAPK family members [i.e. p44/42 (ERK1/2)] activated in adipose tissue during metabolic syndrome, could trigger metaflammation and insulin resistance [3, 4]. It is therefore important to determine whether IL-6 or IL-1β mediates adipose tissue metaflammation during macrophage infiltration via modulating MAPK or NF-κB pathway, to help further elucidate the pathophysiology of metabolic syndrome.

Attenuating adipose tissue metaflammation promotes insulin sensitivity [5], and might be a potential strategy to treat or prevent metabolic syndrome. Accumulated evidence suggests that macrophages play a key role in influencing the proliferation, survival and differentiation of preadipocytes [6–10], which are essential in maintaining adipose tissue homeostasis [11]. Most importantly they might even induce or aggravate metaflammation by stimulating preadipocytes to express and secrete a variety of cytokines, most of which are pro-inflammatory [12–14]. In vitro studies have shown that 1α,25(OH)_2_D_3_ exerts anti-inflammatory actions on lipopolysaccharide (LPS) and macrophage-stimulated adipocytes, by reducing the release and gene expression of major pro-inflammatory factors including IL-6, IL-8 and MCP-1 [15, 16]. However, the anti-inflammatory effects of 1α,25(OH)_2_D_3_ on macrophage-stimulated preadipocytes, remains to be explored in detail.

In this study, we aimed firstly to test whether IL-6 or IL-1β is critical in mediating inflammatory responses in MacCM-stimulated preadipocytes, in terms of pro-inflammatory gene expression and secretion; and secondly to determine the inflammatory signaling pathways activated by examining the phosphorylation of signaling molecules including relA of the NF-κB pathway and p44/42 MAPK. Finally, we investigated whether 1α,25(OH)_2_D_3_, could attenuate the inflammatory responses in MacCM-stimulated preadipocytes, and explored the potential anti-inflammatory mechanisms.

## Methods

### Preparation of MacCM

The human THP-1 monocytic cell line was kindly provided by Professor Helen R Griffiths (Aston University, UK). Monocytes were cultured in T75 flasks in RPMI-1640 medium (Sigma-Aldrich, UK) supplemented with 10% fetal bovine serum and 100 U/ml penicillin, 100 μg/ml streptomycin, and incubated at 37 °C in 95% air and 5% CO_2_. When the monocyte density reached 1 × 10^6^ cells/ml, the differentiation to pro-inflammatory M1 dominant macrophages was induced by 100 nM phorbol 12-myristate 13-acetate (Sigma-Aldrich, UK) for 48 h, and then activated by 1 μg/ml LPS [17] and 1 mM ATP (Sigma-Aldrich, UK) for a further 24 h in RPMI-1640 before medium collection. The MacCM was filtered through 0.22 μm filters and stored at − 80 °C.

### Culture of human white preadipocytes

Human white preadipocytes derived from subcutaneous adipose tissue of a female Caucasian subject (BMI 21; age 44 years) were obtained from PromoCell (Germany). The preadipocytes were cultured in T25 flasks then sub-cultured into 12-well plates (seeding density: 5000 cells per cm^2^) in preadipocyte growth medium (PromoCell, Germany) supplemented with 100 U/ml penicillin, 100 μg/ml streptomycin, and 0.25 μg/ml amphotericin B, and incubated at 37 °C in 95% air and 5% CO_2_.

### MacCM stimulation and blockade of IL-6 and IL-1β action

When confluence was reached, preadipocytes (n = 6 wells per group) were cultured for 24 h in preadipocyte growth medium, which was replaced with 25% MacCM (diluted in RPMI-1640) to elicit inflammatory responses, together with IL-6 antibody (300, 350 and 450 ng/ml) or IL-1β antibody (15 μg/ml as previously established [18]) (R&D Systems, UK) to block IL-6 or IL-1β action by neutralization for a further 24 h before cell and supernatant collection. Isotype control mouse IgG at the same concentrations confirmed that non-specific binding did not block inflammatory responses (data not shown). The control received a mock treatment of 25% preadipocyte growth medium (diluted in RPMI-1640).

### MacCM stimulation and 1α,25(OH)_2_D_3_ pre-treatment and treatment

When confluence was reached, preadipocytes (n = 6 wells per group) were pre-treated with 1α,25(OH)_2_D_3_ (ENZO Life Sciences, USA) (reconstituted in DMSO to a concentration of 1 μg/μl and diluted to final concentrations of 0.01–10 nM in preadipocyte growth medium). The pre-treatment was to boost the potential anti-inflammatory effects of 1α,25(OH)_2_D_3_ as established previously [18]. After 24 h the pre-treatment media were discarded. Then, 25% MacCM (diluted in RPMI-1640) was added together with 1α,25(OH)_2_D_3_ (0.01–10 nM) for a further 24 h before cell and supernatant collection.

### Measurement of inflammatory responses

Cytokine release of the pooled cell supernatants (n = 6) were screened by Human Cytokine Array Panel A following the manufacturer’s instructions (R&D Systems, UK) and Molecular Imager ChemiDoc XRS+ System (Bio-Rad, UK). The results were presented as pixel density relative to the references of the arrays. The secretion levels of major pro-inflammatory factors including IL-1β, IL-6, IL-8, MCP-1 and RANTES were measured in duplicate using human ELISA kits following the manufacturer’s instructions (R&D Systems, UK) and SPECTROstar Nano Microplate Reader (BMG LABTECH, Germany). The results were also normalized to total cell protein content (measured by Thermo Scientific Pierce BCA Protein Assay Kit, UK) and presented as μg(cytokine)/mg(cell protein). The mRNA levels of IL-1β, IL-6, IL-8, MCP-1 and RANTES in preadipocytes were measured in duplicate using TaqMan gene expression assays (Applied Biosystems, UK), qPCR core kit (Eurogentec, Belgium) and Stratagene Mx3005P instrument system. The results were normalized to the values of reference gene PPIA [19] and presented as fold changes of Ct value relative to controls using the 2^−ΔΔct^ formula [20]. The intracellular levels (densities) of inflammatory signaling molecules including relA, phosphorylated relA, p44/42 MAPK, phosphorylated p44/42 MAPK, p38 MAPK and phosphorylated p38 MAPK (New England BioLabs, UK) were measured using western blotting and Molecular Imager ChemiDoc XRS+ System (Bio-Rad, UK). The results were calculated as ratios of phosphorylated relA to relA, phosphorylated p44/42 MAPK to p44/42 MAPK and phosphorylated p38 MAPK to p38 MAPK, normalized to loading control vinculin (Abcam, UK), and presented as fold changes of density relative to controls. All the antibodies used were diluted according to the manufacturer’s instructions. Methods of qPCR and western blotting were as previously described [21].

### Statistical analysis

Results are presented as mean ± SEM. For statistical analysis, unpaired Student’s t test was used for individual group comparisons. One-way ANOVA for independent samples was used, followed by Tukey’s test for individual group comparisons. A value of p < 0.05 was regarded as statistically significant. The analysis and presentation were performed using Prism 5 (GraphPad, USA).

## Results

### IL-6 and IL-1β mediate inflammatory responses in MacCM-stimulated preadipocytes

**Fig. 1:**
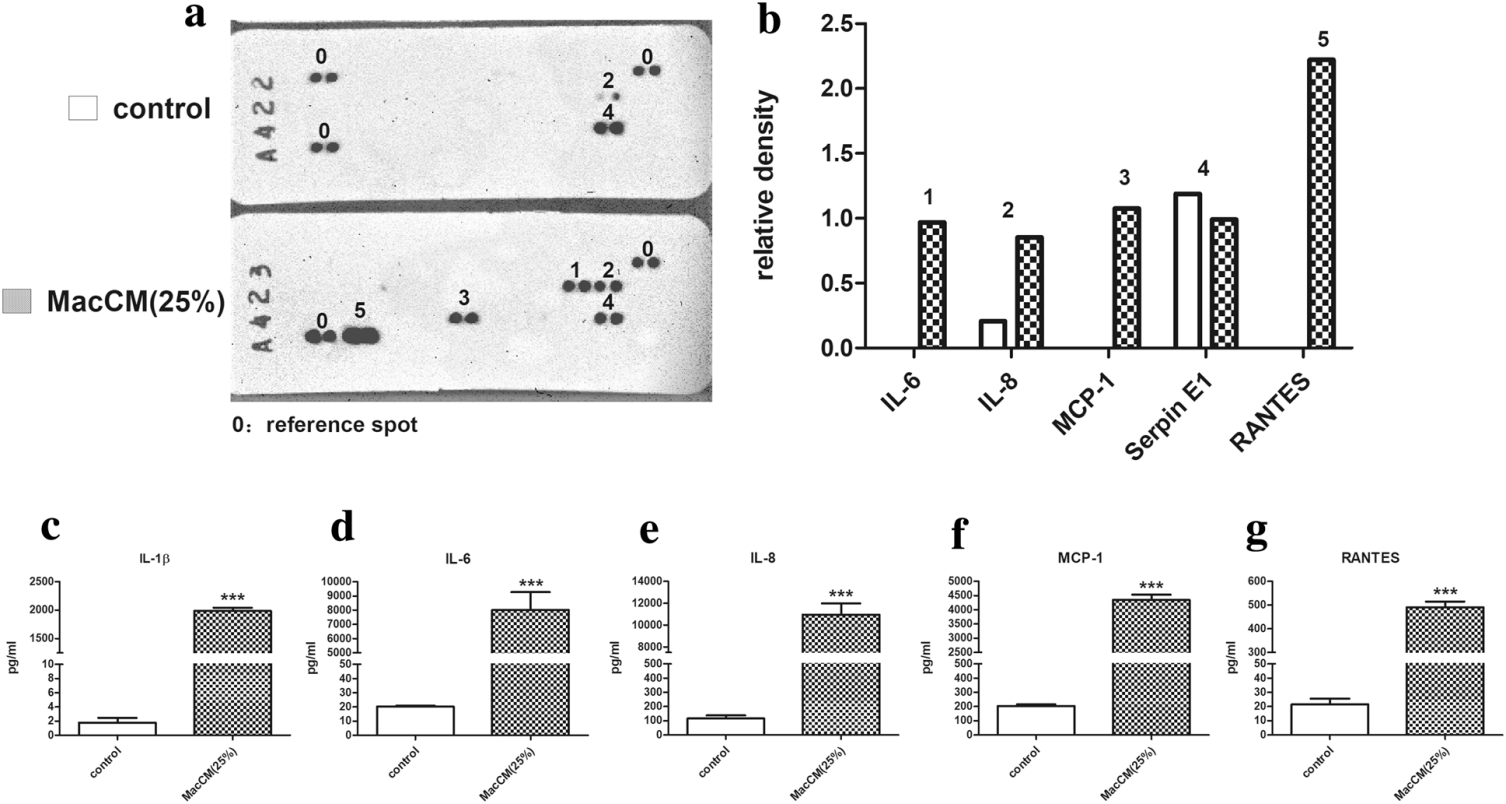
Effects of MacCM on cytokine release from human white preadipocytes. Preadipocytes were either cultured alone (control), or with THP-1-MacCM (25%) for 24 h before supernatant collection. **a** Secreted cytokines were screened by cytokine array. **b** The results are presented as pixel density relative to the reference controls of the arrays. The concentrations of pro-inflammatory factors **c** IL-1β, **d** IL-6, **e** IL-8, **f** MCP-1 and **g** RANTES were also measured by ELISA. Data are shown as mean ± SEM for groups of 6. The results were analyzed using unpaired Student’s t test and confirmed by three independent experiments. A significant difference to control was indicated by ***(p < 0.001)

**Fig. 2:**
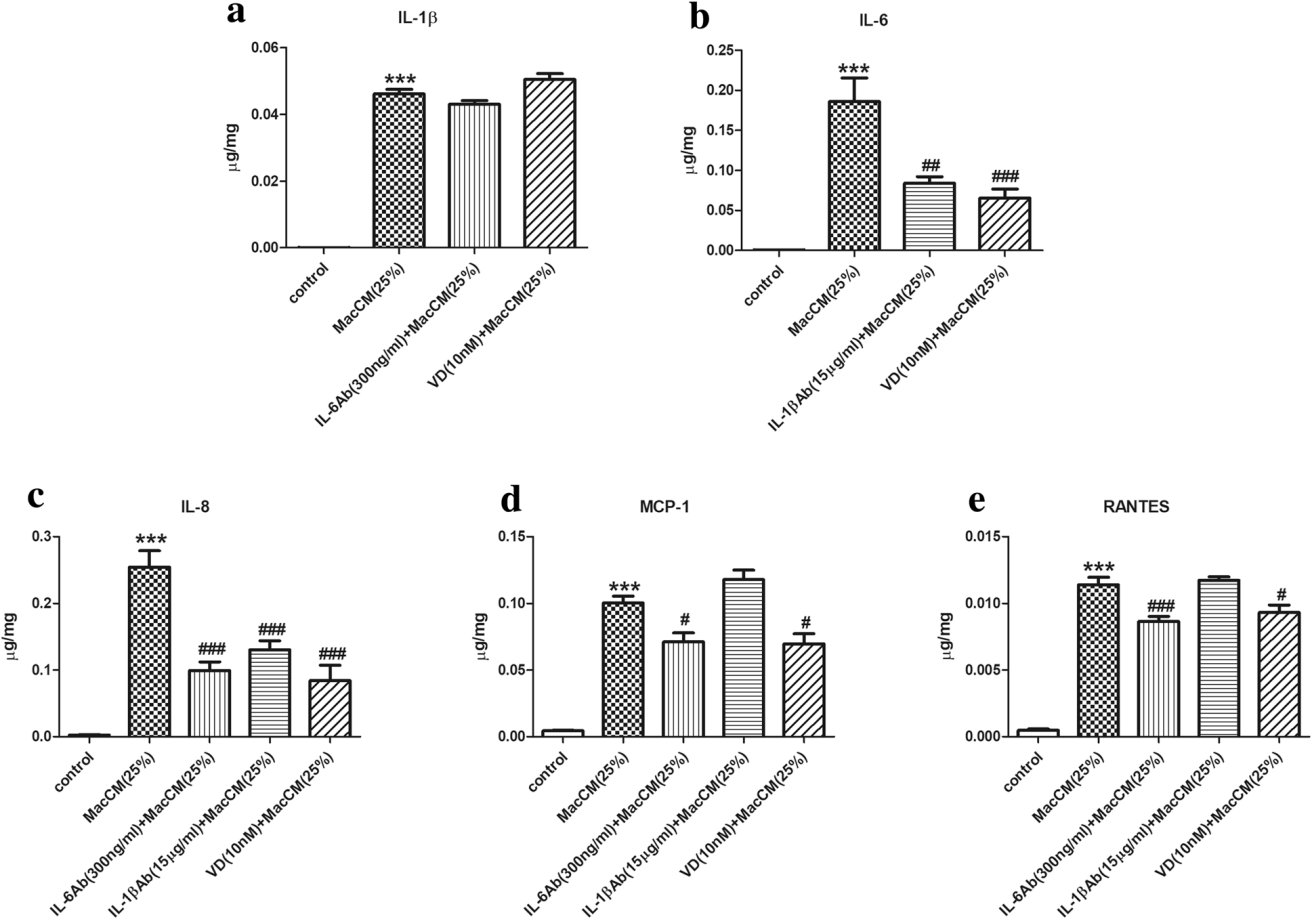
Effects of 1α,25(OH)_2_D_3_ on IL-6 and IL-1β-mediated pro-inflammatory secretion from MacCM-stimulated human white preadipocytes. Preadipocytes were either cultured alone (control), or with THP-1-MacCM (25%), or in the presence of IL-6 antibody (300 ng/ml), or IL-1β antibody (15 μg/ml) for 24 h. A further group of cells was pre-treated with 1α,25(OH)_2_D_3_ (10 nM) for 24 h, followed by treatments with THP-1-MacCM (25%) and 1α,25(OH)_2_D_3_ (10 nM) for a further 24 h before supernatant collection. The release levels of pro-inflammatory factors **a** IL-1β, **b** IL-6, **c** IL-8, **d** MCP-1 and **e** RANTES were measured by ELISA and normalized to total cell protein content. Data are shown as mean ± SEM for groups of 6. The results were analyzed using one-way ANOVA with Tukey’s post hoc test and confirmed by three independent experiments. A significant difference to control was indicated by ***(p < 0.001); to THP-1-MacCM by ^#^(p < 0.05), ^##^(p < 0.01) and ^###^(p < 0.001)

**Fig. 3:**
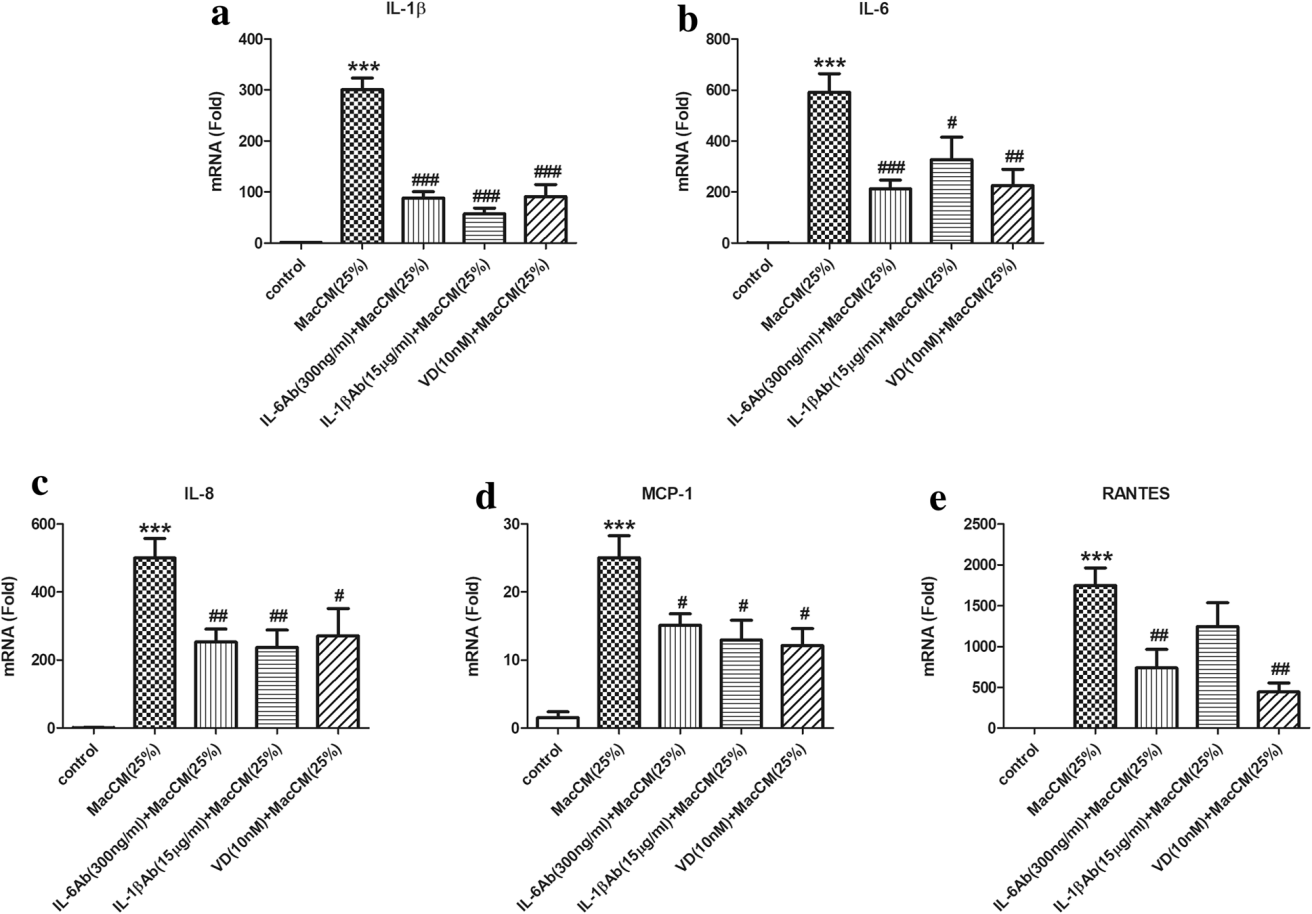
Effects of 1α,25(OH)_2_D_3_ on IL-6 and IL-1β-mediated pro-inflammatory gene expression in MacCM-stimulated human white preadipocytes. Preadipocytes were either cultured alone (control), or with THP-1-MacCM (25%), or in the presence of IL-6 antibody (300 ng/ml), or IL-1β antibody (15 μg/ml) for 24 h. A further group of cells was pre-treated with 1α,25(OH)_2_D_3_ (10 nM) for 24 h, followed by treatments with THP-1-MacCM (25%) and 1α,25(OH)_2_D_3_ (10 nM) for a further 24 h before cell collection. The mRNA levels of pro-inflammatory factors **a** IL-1β, **b** IL-6, **c** IL-8, **d** MCP-1 and **e** RANTES were measured by qPCR. Data are shown as mean ± SEM for groups of 6. The results were analyzed using one-way ANOVA with Tukey’s post hoc test and confirmed by three independent experiments. A significant difference to control was indicated by ***(p < 0.001); to THP-1-MacCM by ^#^(p < 0.05), ^##^(p < 0.01) and ^###^(p < 0.001)

Infiltration of macrophages into adipose tissue induces metaflammation [2], which may contribute to metabolic syndrome [1]. In the initial experiments, we treated human white preadipocytes with MacCM (25%) to elicit inflammatory responses. Cytokine release was screened using proteome array and the results revealed that (Fig. 1a, b) pro-inflammatory factors including IL-6, IL-8, MCP-1 and RANTES were secreted from MacCM-stimulated preadipocytes, compared to controls. Surprisingly, IL-1β, which is vital in initiating and sustaining metaflammation in adipose tissue [22, 23], was not detected. However, (Figs. 1c–g and 2) further ELISA results (unnormalized and normalized) show that besides IL-6, IL-8, MCP-1 and RANTES, the secretion levels of IL-1β were also markedly increased, but relatively lower, possibly too low to be detected by the array. In contrast, the secretion level of serpin was mildly decreased from MacCM-stimulated preadipocytes. Similarly, (Fig. 3) the qPCR results show that the mRNA levels of these pro-inflammatory factors were generally enhanced in MacCM-stimulated preadipocytes, especially IL-1β and IL-6, which were approximately 767 and 404-fold higher, compared to the control.

The plasma level of IL-6 is almost exclusively determined by whole-body adiposity and thus closely associated with metabolic syndrome [14]. Moreover, IL-6 has been found to stimulate the synthesis of C-reactive protein, which is one of the strongest indicators of metabolic risk [24]. Hence, we speculated that IL-6 might mediate inflammatory responses in MacCM-stimulated preadipocytes. To test this, we blocked IL-6 action from MacCM-stimulated preadipocytes using IL-6 antibody. In preliminary work, a range of IL-6 antibody doses were tested based on calculations using the manufacturer’s instructions, and (Additional file 1: Fig. S1PA and S1PB) the results show that 300 ng/ml was the optimal dose to inhibit cytokine secretion and gene expression. In accordance with this, (Fig. 2) the ELISA results (normalized) show that the secretion of IL-8 was considerably decreased by IL-6 antibody, which was 0.6-fold lower compared with MacCM-stimulated preadipocytes. Likewise, the levels of MCP-1 and RANTES were moderately decreased by 0.3- and 0.2-fold, respectively. Although the concentrations of IL-1β were numerically lower, this did not reach statistical significance. The secretion of IL-6 (and IL-1β later) is not presented, since additional IL-6 (IL-1β) antibodies in the supernatant will affect the accuracy of the ELISA results. Moreover, blocking IL-6 action exerted potent inhibitory effects on cytokine gene expression, (Fig. 3) as the mRNA levels of IL-1β, IL-6, IL-8, MCP-1 and RANTES were all significantly lowered by 0.4- to 0.7-fold. Therefore, IL-6 propagates and maintains inflammatory responses during adipose tissue metaflammation, since blocking IL-6 action inhibits the secretion and gene expression of IL-1β, IL-6, IL-8, MCP-1 and RANTES in MacCM-stimulated preadipocytes.

A recent study from our group demonstrated that IL-1β could target preadipocytes to induce adipose tissue metaflammation [25]. Likewise, the current results show that IL-1β is crucial in mediating inflammatory responses in macrophage-stimulated preadipocytes, (Fig. 2) since by blocking IL-1β action from MacCM-stimulated preadipocytes with IL-1β antibody (15 μg/ml), the secretion levels of IL-6 and IL-8 were moderately reduced by 0.5-fold compared with MacCM-stimulated preadipocytes. However, the release of MCP-1 and RANTES were not statistically different in the presence of MacCM compared with the control. In parallel, (Fig. 3) blocking IL-1β action markedly decreased the mRNA levels of IL-1β, IL-6, IL-8, MCP-1 and RANTES by 0.4- to 0.7-fold in MacCM-stimulated preadipocytes.

### IL-6 and IL-1β increase the phosphorylation of inflammatory signaling molecules in MacCM-stimulated preadipocytes

A possible mechanism for induction of adipose tissue metaflammation is activation of the NF-κB signaling pathway via phosphorylation of transcription factor relA [5]. In our study, (Fig. 6b) MacCM considerably increased the phosphorylation levels of relA by 180%, (Fig. 6a) though the levels of NF-κB were similar compared with the control. In accordance with that, the NF-κB signaling pathway could be activated by IL-1β [5], (Fig. 6b) since the decreased phosphorylation levels of relA were significantly associated with blocking IL-1β action.

The conventional MAPKs including p44/42 and p38, are activated through phosphorylation, and are established as playing an important role in various biological processes, especially inflammation [26]. Firstly, (Fig. 6a and Additional file 2: Fig. S2A) compared to the control, MacCM had no effect on the levels of p44/42 MAPK and p38 MAPK. Secondly, (Fig. 6c) the phosphorylation of p44/42 MAPK were moderately increased by 60% in MacCM-stimulated preadipocytes. In parallel, (Additional file 2: Fig. S2B) MacCM remarkably increased the phosphorylation of p38 MAPK, which was 250% higher than observed in the control. Furthermore, it is interesting to note that IL-6 triggers inflammatory responses by activating the p44/42 MAPK signaling pathway [27], as indicated by our observation that the phosphorylation levels of p44/42 MAPK were significantly decreased by blocking IL-6 action in MacCM-stimulated preadipocytes (Fig. 6c).

In addition, (Fig. 6c and Additional file 2: Fig. S2B) show that after antibody blockade of IL-1β, the phosphorylation of p44/42 MAPK and p38 MAPK were slightly lower in MacCM-stimulated preadipocytes, but the difference from control was not statistically significant. Likewise, (Fig. 6b and Additional file 2: Fig. S2B) blocking IL-6 action did not affect the phosphorylation levels of relA or p38 MAPK.

### 1α,25(OH)_2_D_3_ inhibits pro-inflammatory secretion and gene expression in MacCM-stimulated preadipocytes

**Fig. 4:**
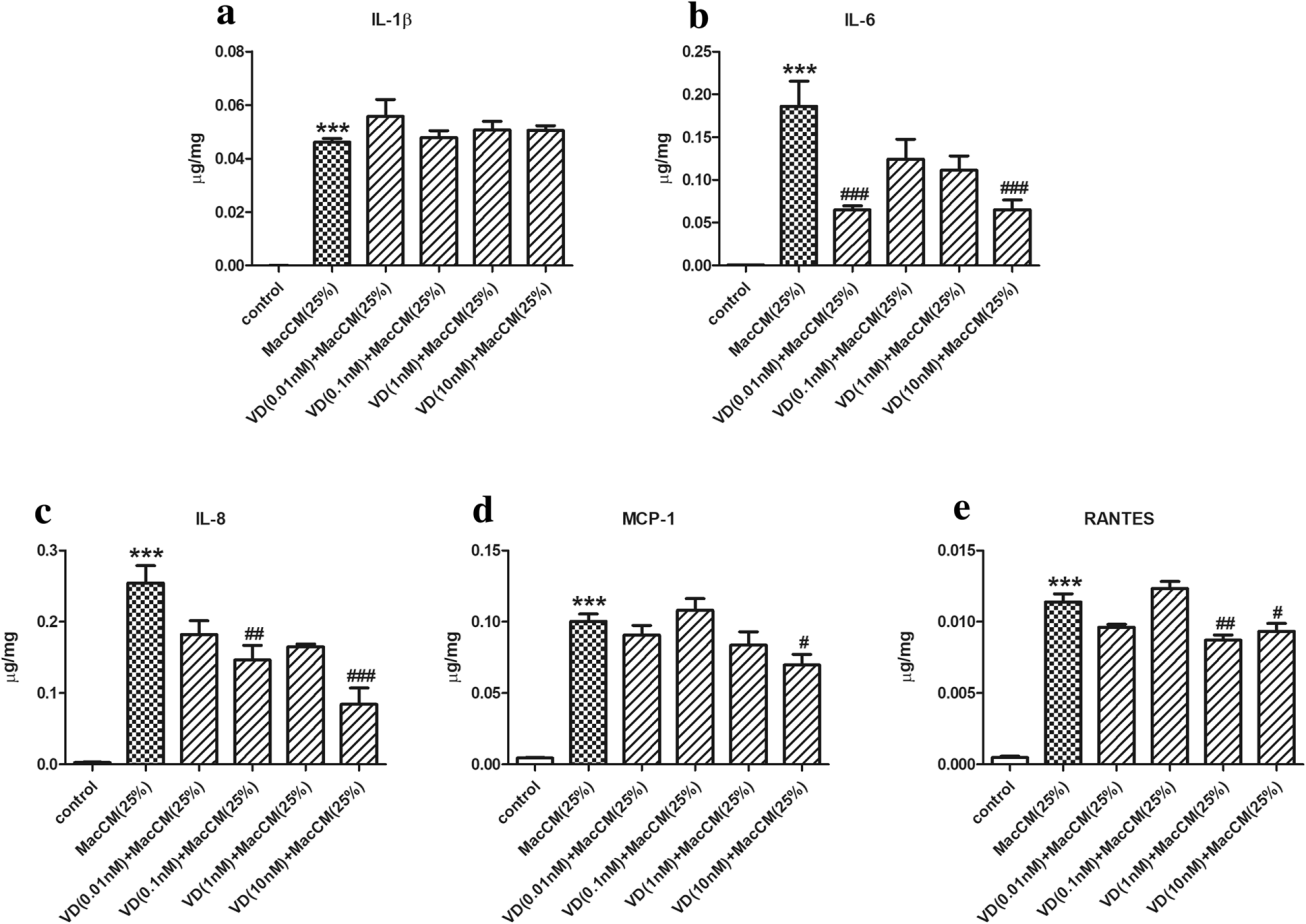
The decreasing effects of 1α,25(OH)_2_D_3_ on pro-inflammatory secretion from MacCM-stimulated human white preadipocytes. Preadipocytes were either cultured alone (control), or with THP-1-MacCM (25%) for 24 h. Further groups of cells were pre-treated with 1α,25(OH)_2_D_3_ (0.01–10 nM) for 24 h, followed by treatments with THP-1-MacCM (25%) and 1α,25(OH)_2_D_3_ (0.01–10 nM) for a further 24 h before supernatant collection. The concentrations of pro-inflammatory factors **a** IL-1β, **b** IL-6, **c** IL-8, **d** MCP-1 and **e** RANTES were measured by ELISA and normalized to total cell protein content. Data are shown as mean ± SEM for groups of 6. The results were analyzed using one-way ANOVA with Tukey’s post hoc test and confirmed by three independent experiments. A significant difference to control was indicated by ***(p < 0.001); to THP-1-MacCM by ^#^(p < 0.05), ^##^(p < 0.01) and ^###^(p < 0.001)

We speculated that 1α,25(OH)_2_D_3_ might attenuate inflammatory responses in MacCM-stimulated preadipocytes, as has previously been demonstrated in IL-1β-stimulated preadipocytes [25]. Initially, MacCM-stimulated preadipocytes were pre-treated and treated with 10 nM of 1α,25(OH)_2_D_3_ (which was a dose formerly established [25]). With respect to pro-inflammatory secretion, (Fig. 2) the ELISA results (normalized) show that compared with MacCM-stimulated preadipocytes, 10 nM of 1α,25(OH)_2_D_3_ markedly decreased the secretion of IL-6 and IL-8 by 0.5- and 0.6-fold, whereas the effects to reduce MCP-1 and RANTES release were moderate (0.3- and 0.2-fold lower, respectively). However, the secretion of IL-1β was not affected in MacCM-stimulated preadipocytes by 10 nM of 1α,25(OH)_2_D_3_. Next, to investigate whether the effects of 1α,25(OH)_2_D_3_ on the inflammatory responses are dose-dependent, we pre-treated and treated MacCM-stimulated preadipocytes with 0.01 to 1 nM of 1α,25(OH)_2_D_3_ using the same methods. With respect to pro-inflammatory secretion, (Fig. 4) firstly, 0.01 to 1 nM of 1α,25(OH)_2_D_3_ did not affect the secretion of IL-1β and MCP-1 in MacCM-stimulated preadipocytes. Secondly, only 0.01 nM of 1α,25(OH)_2_D_3_ dramatically decreased the secretion levels of IL-6 by 0.7-fold, while though numerically lower, no statistically significant differences were seen with other doses. Finally, relatively high doses of 1α,25(OH)_2_D_3_ were significantly associated with reduced levels of IL-8 and RANTES, as observed that 0.1 and 1 nM of 1α,25(OH)_2_D_3_ mildly decreased the secretion levels of IL-8 by 0.4-fold and RANTES by 0.2-fold, respectively.

**Fig. 5:**
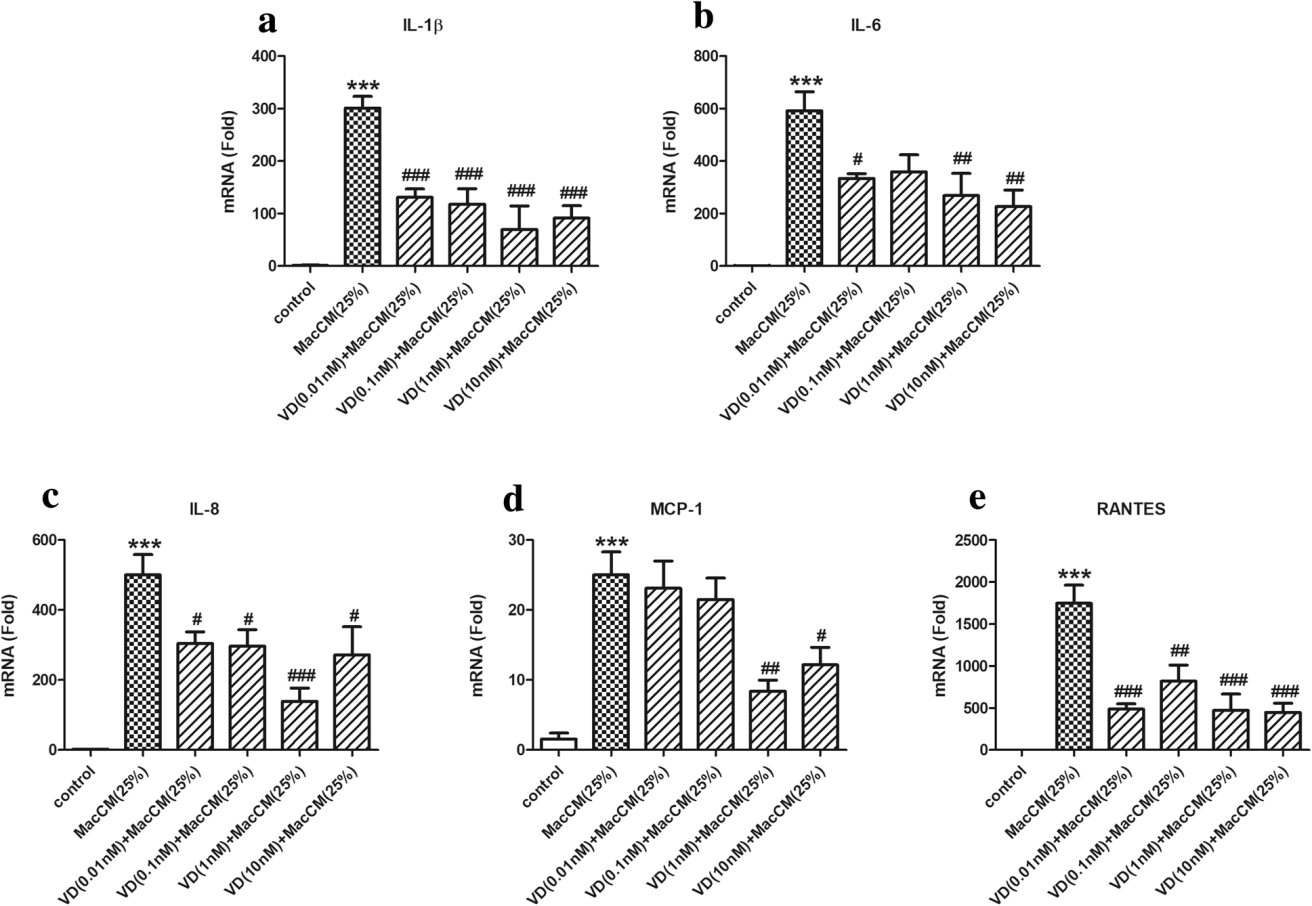
The decreasing effects of 1α,25(OH)_2_D_3_ on pro-inflammatory gene expression in MacCM-stimulated human white preadipocytes. Preadipocytes were either cultured alone (control), or with THP-1-MacCM (25%) for 24 h. Further groups of cells were pre-treated with 1α,25(OH)_2_D_3_ (0.01–10 nM) for 24 h, followed by treatments with THP-1-MacCM (25%) and 1α,25(OH)_2_D_3_ (0.01–10 nM) for a further 24 h before cell collection. The mRNA levels of pro-inflammatory factors **a** IL-1β, **b** IL-6, **c** IL-8, **d** MCP-1 and **e** RANTES were measured from cells by qPCR. Data are shown as mean ± SEM for groups of 6. The results were analyzed using one-way ANOVA with Tukey’s post hoc test and confirmed by three independent experiments. A significant difference to control was indicated by ***(p < 0.001); to THP-1-MacCM by ^#^(p < 0.05), ^##^(p < 0.01) and ^###^(p < 0.001)

In parallel, 1α,25(OH)_2_D_3_ exerted complex, but in general inhibitory effects on pro-inflammatory gene expression in MacCM-stimulated preadipocytes. As shown in Figs. 3 and 5, 0.01 to 10 nM of 1α,25(OH)_2_D_3_ inhibited the mRNA levels of IL-1β and RANTES by 0.5- to 0.8-fold. The mRNA levels of IL-8 were reduced 0.7-fold by 1 nM of 1α,25(OH)_2_D_3_; other doses had similar but moderate inhibitory effects on IL-8 gene expression. The mRNA levels of IL-6 were decreased by 0.4- to 0.6-fold, by all doses except 0.1 nM of 1α,25(OH)_2_D_3_, which had no effect. However, only 1 and 10 nM of 1α,25(OH)_2_D_3_ reduced mRNA levels of MCP-1 by 0.7- and 0.5-fold, respectively.

Therefore, 1α,25(OH)_2_D_3_ attenuates IL-1β and IL-6-mediated inflammatory responses in MacCM-stimulated preadipocytes. In addition, (Figs. 2, 3) 10 nM of 1α,25(OH)_2_D_3_ generally reduced pro-inflammatory secretion and gene expression, (Figs. 4, 5) but there was no consistent dose–response relationship between the pro-inflammatory secretion or gene expression and the doses of 1α,25(OH)_2_D_3_ used in our study.

### 1α,25(OH)_2_D_3_ decreases the phosphorylation of relA of the NF-κB signaling pathway and p44/42 MAPK in MacCM-stimulated preadipocytes

**Fig. 6:**
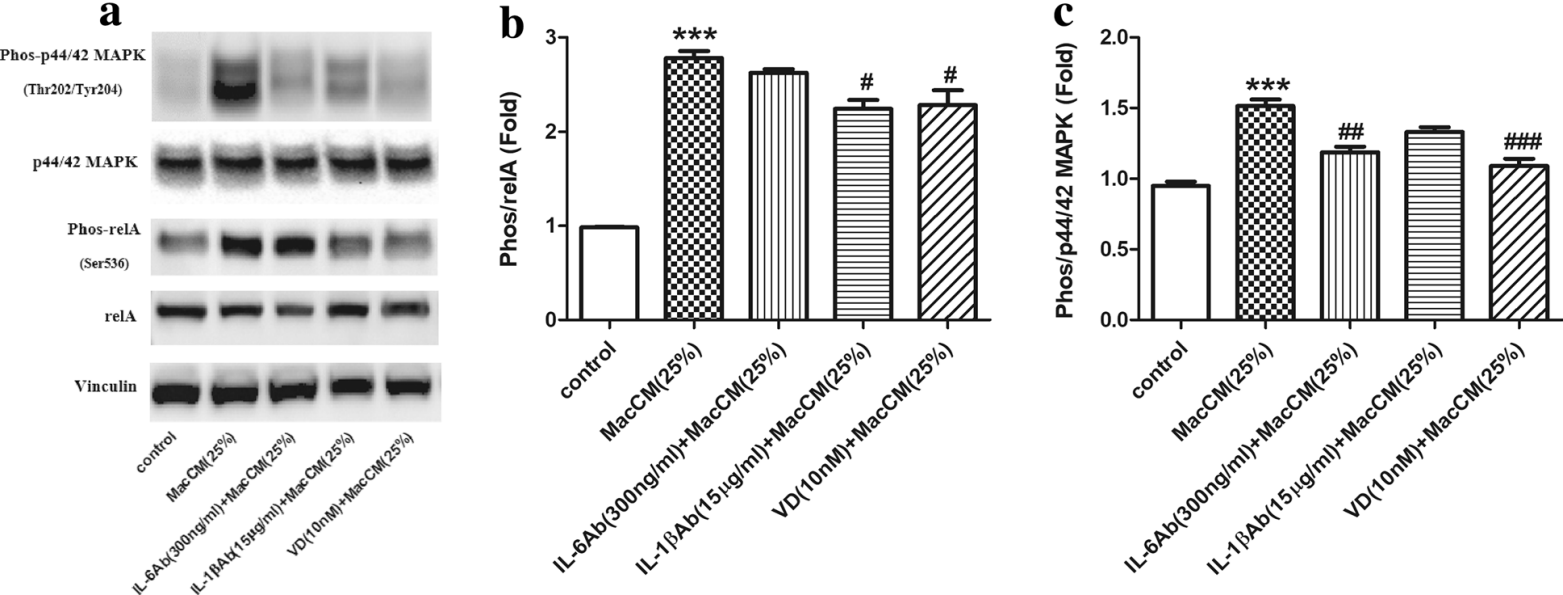
Modifying effects of IL-6, IL-1β and 1α,25(OH)_2_D_3_ on the phosphorylation of relA and p44/42 MAPK in MacCM-stimulated human white preadipocytes. Preadipocytes were either cultured alone (control), or with THP-1-MacCM (25%), or in the presence of IL-6 antibody (300 ng/ml), or IL-1β antibody (15 μg/ml) for 24 h. A further group of cells was pre-treated with 1α,25(OH)_2_D_3_ (10 nM) for 24 h, followed by treatments with THP-1-MacCM (25%) and 1α,25(OH)_2_D_3_ (10 nM) for a further 24 h before lysate collection. **a** The levels of relA, phosphorylated relA, p44/42 MAPK and phosphorylated p44/42 MAPK were measured by western blotting. The results are presented as fold changes of ratios of **b** phosphorylated relA to relA, **c** phosphorylated p44/42 MAPK to p44/42 MAPK to controls. Data are shown as mean ± SEM for groups of 6. The results were analyzed using one-way ANOVA with Tukey’s post hoc test and confirmed by three independent experiments. A significant difference to control was indicated by ***(p < 0.001); to THP-1-MacCM by ^#^(p < 0.05), ^##^(p < 0.01) and ^###^(p < 0.001)

10 nM of 1α,25(OH)_2_D_3_ had no effect on the levels of relA (Fig. 6a), but moderately decreased the phosphorylation levels of relA by 20%, compared to MacCM-stimulated preadipocytes (Fig. 6b). Similarly, (Fig. 6a) the levels of p44/42 MAPK were not affected by 10 nM of 1α,25(OH)_2_D_3_, but their phosphorylation was reduced by 30% (Fig. 6c). Although (Additional file 2: Fig. S2) 10 nM of 1α,25(OH)_2_D_3_ had no effect on the levels of p38 MAPK, those of phosphorylated p38 MAPK were not affected, either. Additionally, lower doses of 1α,25(OH)_2_D_3_ (0.01 to 1 nM) had no effects on any of the above signaling pathways (data not shown).

Overall, 1α,25(OH)_2_D_3_ decreases the phosphorylation of inflammatory signaling molecules including relA and p44/42 MAPK in MacCM-stimulated preadipocytes, but only 10 nM of 1α,25(OH)_2_D_3_ exerted such effects in MacCM-stimulated preadipocytes.

## Discussion

The initial results show that in human white preadipocytes, MacCM massively enhanced the secretion and gene expression of major pro-inflammatory factors including IL-1β, IL-6, IL-8, MCP-1 and RANTES, which have been measured as indicators of adipose tissue metaflammation in keeping with published studies [14, 24, 28–31]. Although downstream markers of adipose tissue inflammation such as leptin and adiponectin are of interest [32], these adipokines are usually secreted by mature adipocytes; preadipocytes express and secrete extremely low levels of adiponectin and leptin, which we did test in this study (data not shown). Interestingly, we also found that TNF-α, one of the major pro-inflammatory factors released from adipocytes in metaflammation [4], was not secreted at detectable levels from MacCM-stimulated preadipocytes. Hence, it appears that preadipocytes have a unique secretome compared to mature adipocytes during metaflammation.

Considered as an adipokine [14], IL-6 is also a multifaceted, pleiotropic cytokine and suggested to play a central role in the development of metabolic syndrome by inducing metaflammation and insulin resistance [33]. Hence, rather than neutralizing IL-1β in MacCM as formally established [18], we blocked IL-6 and IL-1β action during the stimulation, to determine whether either of them could mediate the inflammatory responses in MacCM-stimulated preadipocytes. The present study showed that both IL-6 and IL-1β mediate MacCM-stimulated inflammatory responses in preadipocytes by increasing the phosphorylation of relA of the NF-κB pathway and p44/42 MAPK, respectively. Moreover, it might be suggested that IL-6 exerts broader inflammatory effects on preadipocytes, for most of the pro-inflammatory factors (though not IL-1β) were inhibited when IL-6 action was blocked, whilst the chemoattractants MCP-1 and RANTES were not significantly reduced by IL-1β blockade. However, it is clear that IL-1β is also important as its blockade did significantly attenuate the inflammatory responses.

**Fig. 7:**
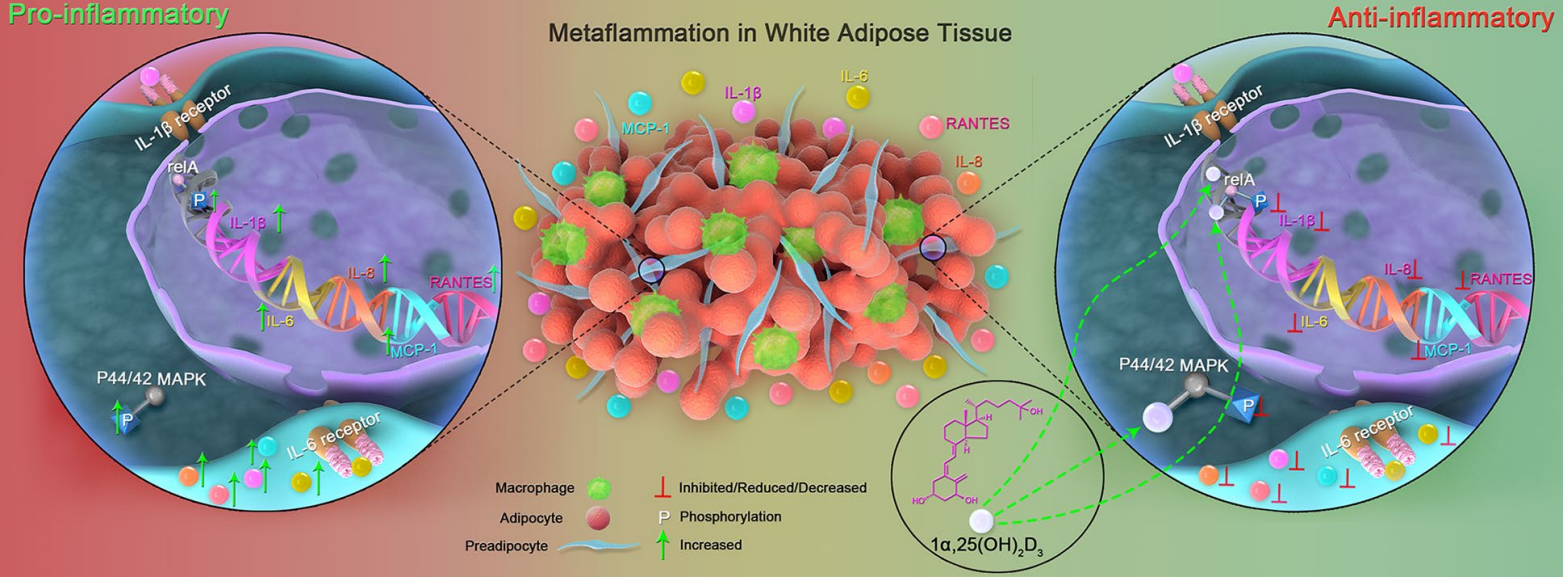
1α,25(OH)_2_D_3_ attenuates Il-6 and IL-1β-mediated inflammatory responses in MacCM-stimulated human white preadipocytes. Accumulated macrophages infiltrating into white adipose tissue induce metaflammation, which is embodied by inflammatory responses including increased local gene expression and secretion of pro-inflammatory factors, particularly IL-1β, IL-6, IL-8, MCP-1 and RANTES. Among these pro-inflammatory factors, by binding to its receptor, IL-1β initiates and sustains the inflammatory responses in macrophage-stimulated preadipocytes by enhancing the phosphorylation of relA of the NF-κB signaling pathway, while in the same ligand-receptor manner IL-6 mediates similar responses by enhancing the phosphorylation of p44/42 MAPK. Besides directly inhibiting the pro-inflammatory gene expression (not tested in our study), 1α,25(OH)_2_D_3_ attenuates IL-1β-mediated inflammatory responses by decreasing the phosphorylation of relA in the nucleus. Moreover, 1α,25(OH)_2_D_3_ can exert a non-genomic action by decreasing the phosphorylation of p44/42 MAPK in the cytoplasm, to attenuate IL-6-mediated inflammatory responses

Intriguingly, our results suggest that 1α,25(OH)_2_D_3_ could attenuate inflammatory responses in macrophage-stimulated preadipocytes during adipose tissue metaflammation, for 1α,25(OH)_2_D_3_ significantly inhibited pro-inflammatory secretion from MacCM-stimulated preadipocytes. Furthermore, 1α,25(OH)_2_D_3_ inhibits the mRNA of the major pro-inflammatory factors in MacCM-stimulated preadipocytes probably by decreasing phosphorylation of relA, which is consistent with other relevant studies that phosphorylated (activated) relA enhances the expression of pro-inflammatory genes including IL-1β, IL-6 and MCP-1 [34]. It is also possible that 1α,25(OH)_2_D_3_ could inhibit pro-inflammatory gene expression by antagonizing the action of relA in the nucleus [35, 36]. Besides the genomic effects, 1α,25(OH)_2_D_3_ can exert non-genomic actions in the cytoplasm by modulating the MAPK pathways [37], which we also found, as 1α,25(OH)_2_D_3_ might inhibit the pro-inflammatory responses by reducing p44/42 MAPK phosphorylation in MacCM-stimulated preadipocytes. Possible limitations of our study are that we did not test whether 1α,25(OH)_2_D_3_ could directly modulate the pro-inflammatory gene expression in the nucleus by chromatin immunoprecipitation, as 1α,25(OH)_2_D_3_ inhibits the expression of genes including IL-2, IL-12, TNF-α, IFN-γ, GM-CSF, which are important in attenuating inflammatory responses in various tissues [38–41]. In addition, since the anti-inflammatory properties of 1α,25(OH)_2_D_3_ could be exerted in MacCM-stimulated preadipocytes in single or multiple above-mentioned manners (Fig. 7), which might explain why there was no obvious dose–response relationship between 1α,25(OH)_2_D_3_ and the pro-inflammatory secretion or gene expression.

A body of evidence has revealed that metaflammation might be a critical link between metabolic syndrome and cardiovascular disease [42]. Our results suggest that vitamin D might be an independent protective factor to cardiovascular risk due to its anti-inflammatory properties, and given that it also influences several phases of the atherosclerotic process, especially influencing vascular remodeling and atherothrombosis [43]. Moreover, given the benefits of canakinumab seen in the CANTOS trial [44], along with the present findings, IL-1β blocker and 1α,25(OH)_2_D_3_ might complete each other to attenuate metaflammation in adipose tissue, thus to potentially help prevent metabolic syndrome and subsequent cardiovascular disease.

## Conclusions

1α,25(OH)_2_D_3_ attenuates IL-6 and IL-1β-mediated inflammatory responses, probably by inhibiting p44/42 and relA phosphorylation in MacCM-stimulated human white preadipocytes.

## Additional files

**Additional file 1: Figure S1.** IL-6 antibodies block inflammatory responses in MacCM-stimulated human white preadipocytes. Preadipocytes were either cultured alone (control), with THP-1-MacCM (25%), or in the presence of IL-6 antibody (300, 350 and 450 ng/ml) for 24 h before supernatant and cell collection. (Panel A) The release levels of pro-inflammatory factors (A) IL-1β, (B) IL-8, (C) MCP-1 and (D) RANTES were measured by ELISA and normalized to total cell protein content. (Panel B) The mRNA levels of pro-inflammatory factors (A) IL-1β, (B) IL-6, (C) IL-8, (D) MCP-1 and (E) RANTES were measured by qPCR. Data are shown as means ± SEM for groups of 6. The results were analyzed using one-way ANOVA with Tukey’s post hoc test and confirmed by three independent experiments. A significant difference to control was indicated by ***(p<0.001); to THP-1-MacCM by #(p<0.05), ##(p<0.01) and ###(p<0.001).

**Additional file 2: Figure S2.** Modifying effect of MacCM on the phosphorylation of p38 MAPK in human white preadipocytes. Preadipocytes were either cultured alone (control), with THP-1-MacCM (25%), or in the presence of IL-6 antibody (300 ng/ml), or IL-1β antibody (15 μg/ml) for 24 h. A further group of cells was pre-treated with 1α,25(OH)_2_D_3_ (10 nM) for 24 h, followed by treatments with THP-1-MacCM (25%) and 1α,25(OH)_2_D_3_ (10 nM) for a further 24 h before lysate collection. The p38 MAPK and phosphorylated p38 MAPK were measured by western blotting. The results are presented as fold changes of ratios of phosphorylated 38 MAPK to p38 MAPK to controls. Data are shown as means ± SEM for groups of 6. The results were analyzed using one-way ANOVA with Tukey’s post hoc test and confirmed by three independent experiments. A significant difference to control was indicated by ***(p<0.001).

## Abbreviations

MAPK: mitogen-activated protein kinase
IL: interleukin
MacCM: macrophage conditioned medium
MCP-1: monocyte chemoattractant protein-1
RANTES: regulated on activation, normal T cell expressed and secreted
LPS: lipopolysaccharide
ATP: adenosine triphosphate
