# Supplementary figures and images for: 1α,25(OH)2D3 attenuates IL-6 and IL-1β-mediated inflammatory responses in macrophage conditioned medium-stimulated human white preadipocytes by modulating p44/42 MAPK and NF-κB signaling pathways
Source: Diabetol Metab Syndr. 2019 Jan 25;11:9. doi: 10.1186/s13098-019-0405-2 (PMC6346557; doi:10.1186/s13098-019-0405-2)

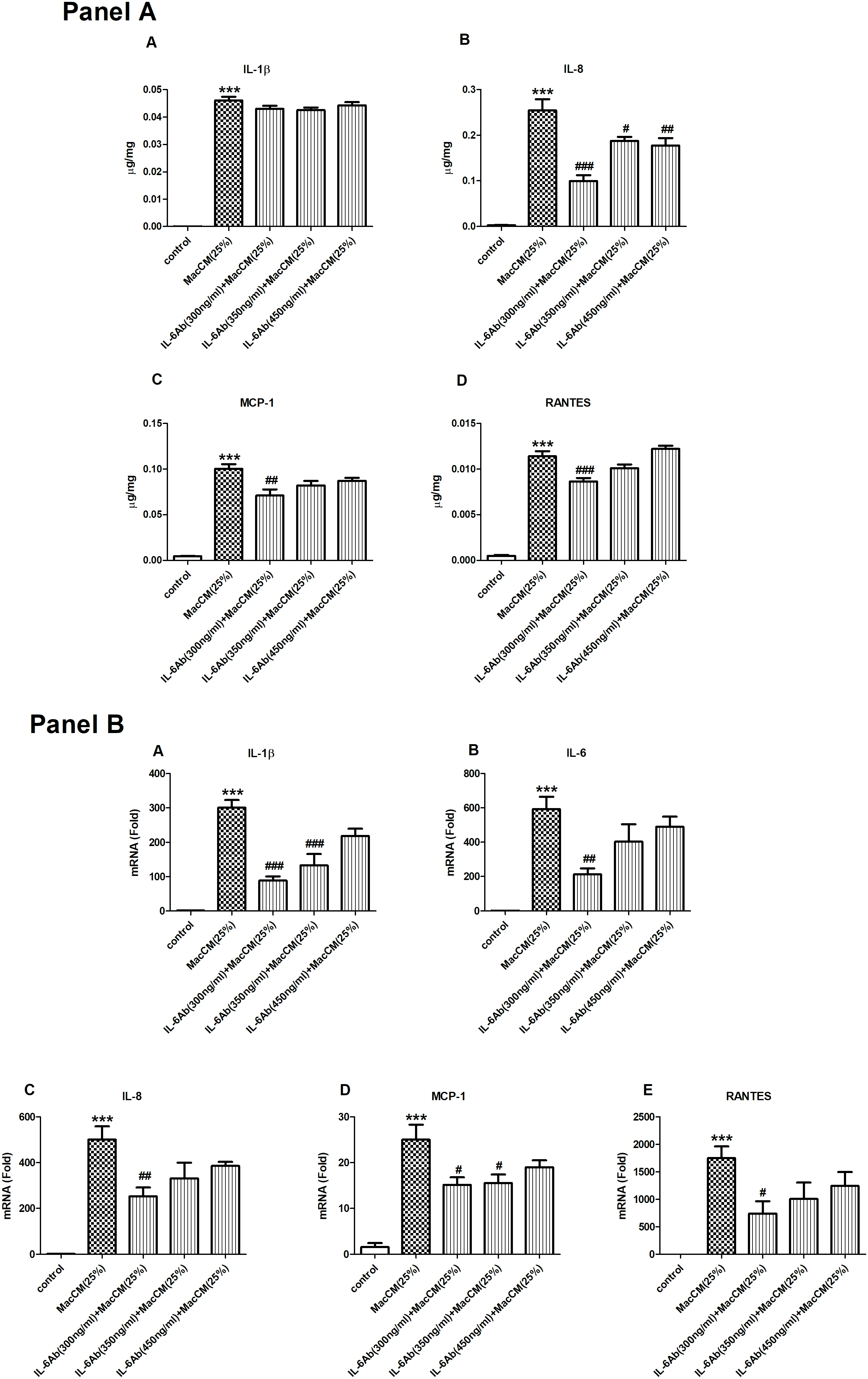

Supplement: Supplementary file 1 — Additional file 1: Figure S1. IL-6 antibodies block inflammatory responses in MacCM-stimulated human white preadipocytes. Preadipocytes were either cultured alone (control), with THP-1-MacCM (25%), or in the presence of IL-6 antibody (300, 350 and 450 ng/ml) for 24 h before supernatant and cell collection. (Panel A) The release levels of pro-inflammatory factors (A) IL-1β, (B) IL-8, (C) MCP-1 and (D) RANTES were measured by ELISA and normalized to total cell protein content. (Panel B) The mRNA levels of pro-inflammatory factors (A) IL-1β, (B) IL-6, (C) IL-8, (D) MCP-1 and (E) RANTES were measured by qPCR. Data are shown as means ± SEM for groups of 6. The results were analyzed using one-way ANOVA with Tukey’s post hoc test and confirmed by three independent experiments. A significant difference to control was indicated by ***(p<0.001); to THP-1-MacCM by #(p<0.05), ##(p<0.01) and ###(p<0.001). [file 13098_2019_405_MOESM1_ESM.tif]

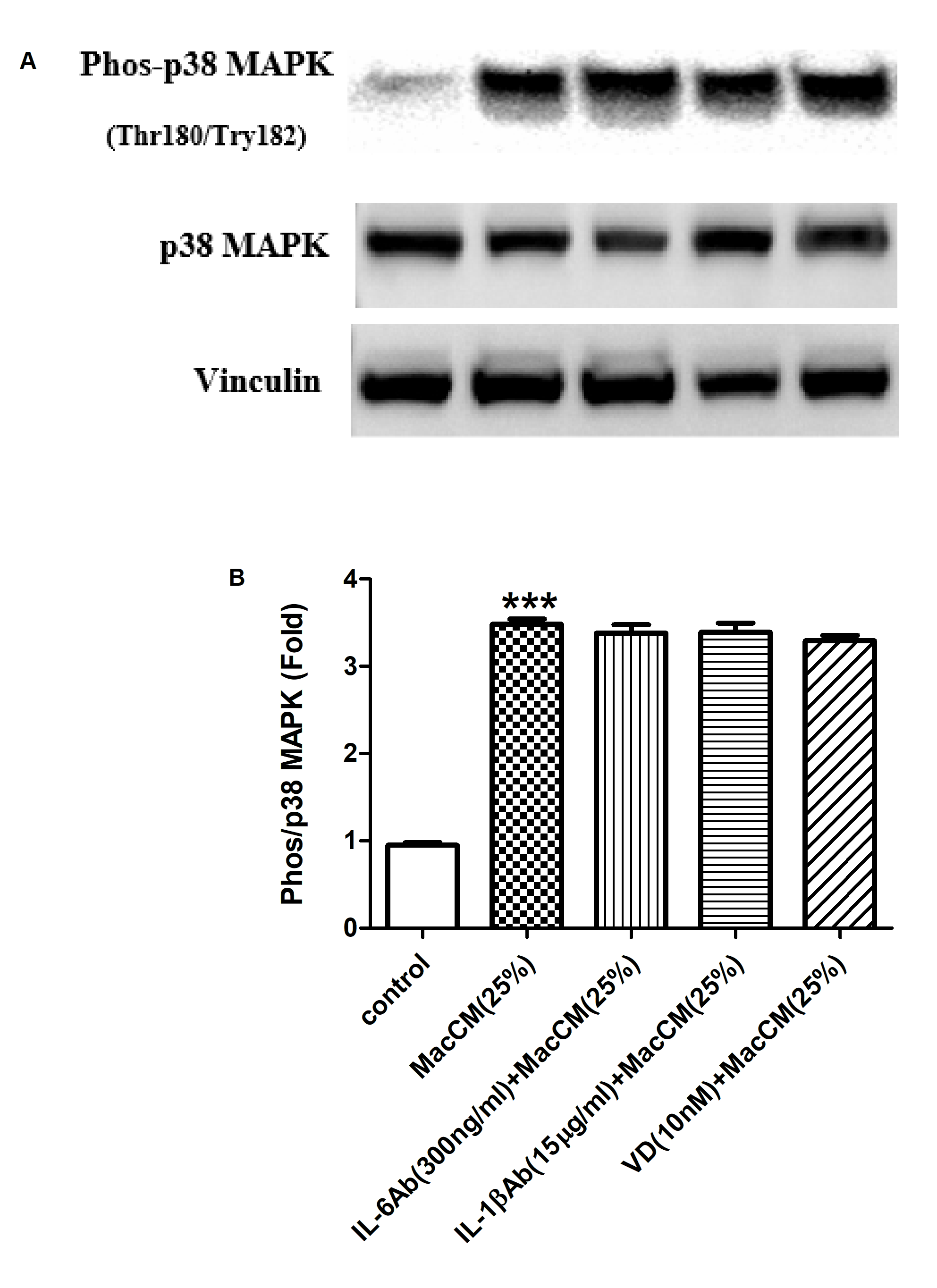

Supplement: Supplementary file 2 — Additional file 2: Figure S2. Modifying effect of MacCM on the phosphorylation of p38 MAPK in human white preadipocytes. Preadipocytes were either cultured alone (control), with THP-1-MacCM (25%), or in the presence of IL-6 antibody (300 ng/ml), or IL-1β antibody (15 μg/ml) for 24 h. A further group of cells was pre-treated with 1α,25(OH)2D3 (10 nM) for 24 h, followed by treatments with THP-1-MacCM (25%) and 1α,25(OH)2D3 (10 nM) for a further 24 h before lysate collection. The p38 MAPK and phosphorylated p38 MAPK were measured by western blotting. The results are presented as fold changes of ratios of phosphorylated 38 MAPK to p38 MAPK to controls. Data are shown as means ± SEM for groups of 6. The results were analyzed using one-way ANOVA with Tukey’s post hoc test and confirmed by three independent experiments. A significant difference to control was indicated by ***(p<0.001). [file 13098_2019_405_MOESM2_ESM.tif]
